# P-933. Daptomycin Resistant Enterococci - An Antibiotic Stewardship Crisis

**DOI:** 10.1093/ofid/ofaf695.1136

**Published:** 2026-01-11

**Authors:** Jeremy Levin, Debra Chew, Nadeem Baalbaki

**Affiliations:** Rutgers- New Jersey Medical School, Bloomfield, NJ; Rutgers New Jersey Medical School, Newark, NewJersey; University Hospital, Newark, NJ

## Abstract

**Background:**

Daptomycin-resistant *Enterococcus faecium* (DRE) is a growing public health concern given the limited therapeutic options available. We sought to investigate risk factors and patient outcomes associated with DRE.

**Figure:**
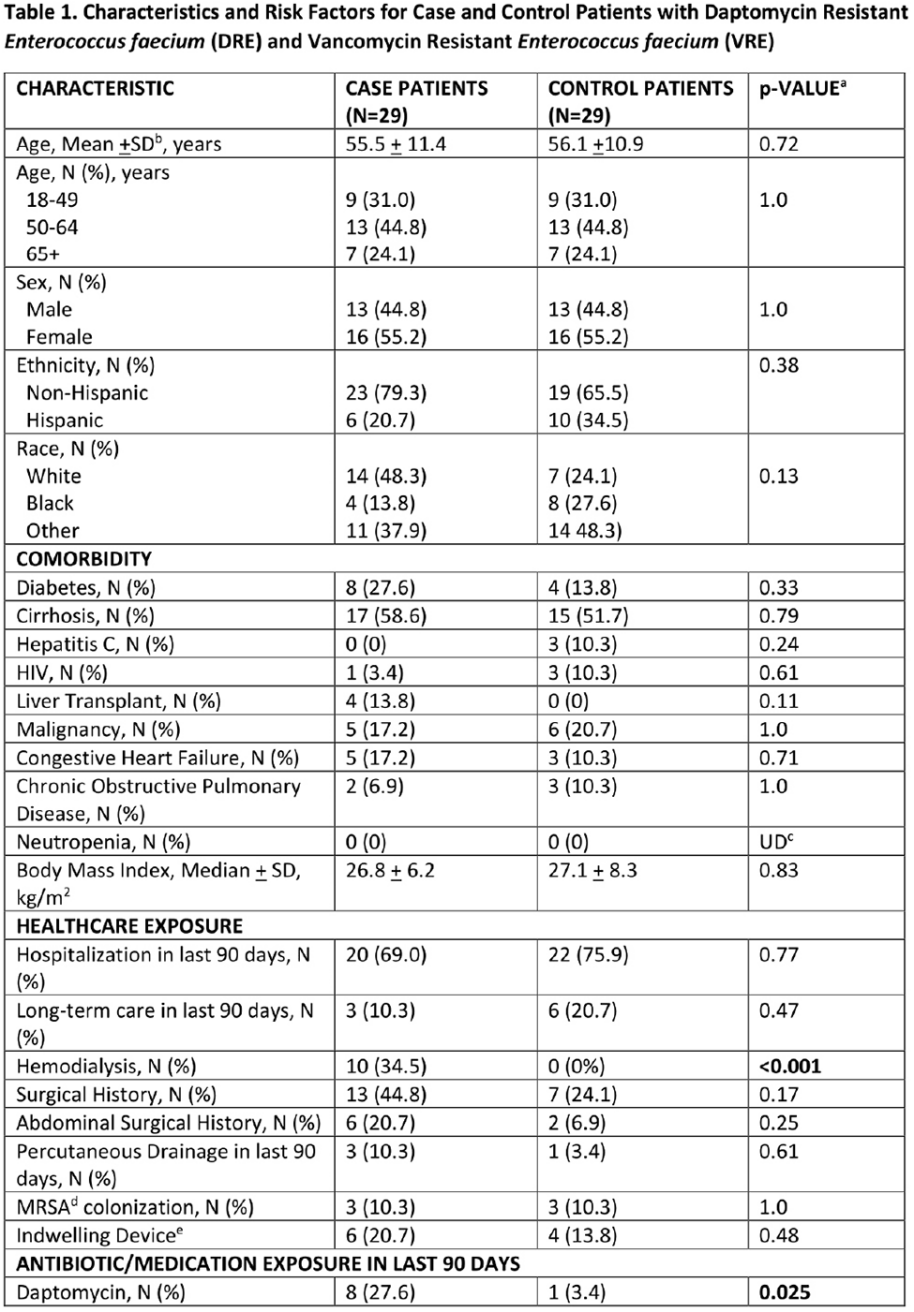

**Figure:**
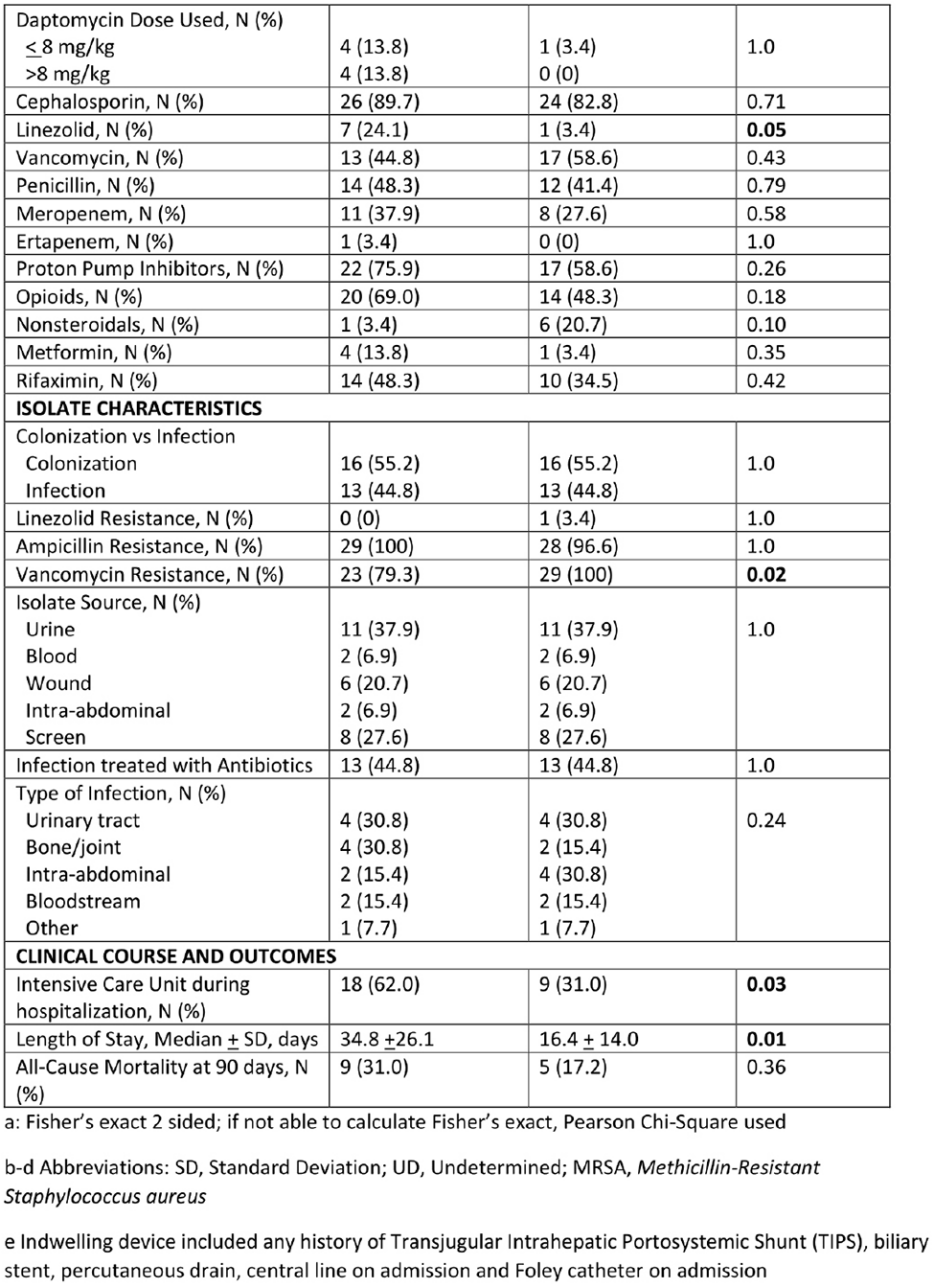

**Methods:**

We conducted a retrospective review of patients with DRE and Vancomycin-resistant *Enterococcus faecium* (VRE) at our institution from 2023 to 2024. Cases were defined as unduplicated patients with a daptomycin minimum inhibitory concentration of >4 ug/mL. Controls were selected from patients with a non-duplicate VRE isolate. For both cases and controls, only the first isolate for each patient in that particular calendar year was included. Controls were matched 1:1 to cases by age (± 5 years), sex at birth, specimen source, and infection versus colonization status.

**Figure:**
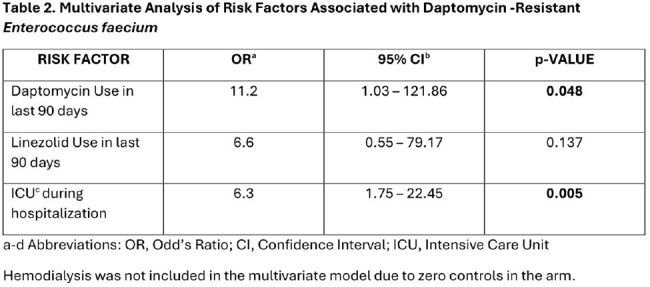

**Results:**

A total of 29 DRE cases were identified and matched with 29 VRE controls. Baseline characteristics and risk factors for patients with DRE and VRE are shown in Table 1. Patients with DRE were more likely than controls to have a history of hemodialysis, exposure to daptomycin and linezolid in the last 90 days, intensive care unit (ICU) stay during their hospitalization, and longer length of stay (LOS). In the multivariate analysis (see Table 2), ICU stay during hospitalization (OR 6.3, p< .01) and exposure to daptomycin in the last 90 days (OR 11.2, p=.048) were significant predictors in patients with DRE.

**Conclusion:**

In our review, daptomycin exposure is associated with an increased risk of acquisition for DRE. Patients with DRE also had worse outcomes, including ICU stay and an increased LOS compared to patients with VRE. These findings display the importance of antibiotic stewardship in limiting unnecessary daptomycin exposure in mitigating the risk of DRE acquisition.

**Disclosures:**

All Authors: No reported disclosures

